# Cases in Medical and Surgical Practice

**Published:** 1860-03

**Authors:** A. Lopez

**Affiliations:** Mobile, Alabama


ART. II.—*Cases in Medical and Surgical Practice.* By A. LOPEZ, M.D., of Mobile, Alabama.

CASE I. *Opium an Antidote to Belladonna.*—W. M. B., apothecary at the U. S. Marine Hospital, had suffered for many months with chronic inflammation of the bursa in the right knee, accompanied by enlargement, and occasionally by great pain. Various remedial measures afforded but temporary and trifling relief. Of his own accord, he applied a large blister on and around the swollen joint, and, after removing the cuticle, a plaster of extract of belladonna to the entire denuded surface. In less than an hour toxicosis was established, as denoted by muscular depression, giddiness, nausea, and extensive dilatation of the pupils; the face, breast, and thighs were suffused with a bright erythema accompanied by intolerable itching; and there existed dry, harsh constriction of fauces and throat, and great thirst. At this stage of things I arrived at the hospital on my morning visit.

Having, on various occasions, successfully tried the use of belladonna in cases of poisoning by opium, as recommended by Dr. Joseph Anderson,* I was induced to test the reciprocal influential relation between the two poisons, and prescribed forthwith Tinct. Opii f℥j, Aq. Cinnamon ℥j, at one dose, with directions to repeat gtts. xv every half hour, until a decided abatement of the pathological symptoms. The first dose antagonized the belladonna in less than thirty minutes after it was taken. The dose of fifteen drops was taken, and the desired result having been obtained at the period prescribed, it was suspended.

CASE II. *Kousso in the Treatment of Tape-worm.*—William Nottingham, English seaman, was admitted into the Marine Hospital on the evening of 26th November, 1855, laboring under all the symptoms of tape-worm. He first discovered its presence while on the eastern coast of Africa, twenty months previously, and had, at various times since, passed very small fragments.

The treatment I pursued was as follows:—

27th.—Thin soup diet.

28th.—Seidlitz powder at bedtime.

29th, 7 A.M.—Kousso, ℥ss; Aq. bullient, ℥xij. Infuse fifteen minutes, cool, strain, and give at a draught, to be followed in one hour by a cup

* See Proceedings of Society of Edinburgh, Monthly Jour. Med. Sci., April, 1854. The suggestion was derived from the practice of Dr. Graves, of Dublin, who used it for the dilatation of the pupils, in order to relieve cerebral derangement of continued fever, etc.

of warm tea. At eleven o'clock, a Seidlitz powder. At 2 P.M., he passed eighteen feet, unbroken, of the worm.

Satisfied that this was not all, I resolved to repeat the remedy, but he left without permission before my next visit.

CASE III. *Rupture of the Spleen; Death.*—Charles Boyer, from the barque Ocilla, was admitted into U. S. Marine Hospital, on the 7th of May, 1856, in *articulo mortis.* He was reported to have received an injury on his back and shoulders by the fall of a large and heavy box of merchandise from a height of about fourteen feet.

On examination, I discovered slight ecchymosis over each scapula, and a very extensive effusion in the left dorso-lumbar region, just about the region of the spleen. He was speechless when brought to the ward, and died in a few hours.

*Autopsy.*—The abdominal cavity contained nearly one gallon of fluid blood with a large quantity of coagula. The inferior and posterior portions of the diaphragm were much bruised and ecchymosed. The spleen was ruptured obliquely throughout its entire length, and was remarkably friable.

CASES IV. and V. *Transverse Fracture of Patella.*—Two cases of this troublesome accident were treated at the Marine Hospital, in 1855, with satisfactory result, by means of Amesbury's apparatus, as described in Plate 5, Fig. 1, of "*Surgery Illustrated.* By SIDNEY DOANE."

The first case was produced by a fall, bringing the patella in sudden and violent contact with a large stone. The second resulted from a blow. In both, the space between the separated portions was extreme, the greatest being nearly two inches. When my patients were discharged, the union restored the patellæ so closely to their original adaptation as to leave scarcely the one-sixteenth of an inch interspace, and scarcely an appreciable alteration in the gait. Having tried on former occasions other methods, I decidedly prefer Amesbury's apparatus.

CASE VI. *Milky Urine.*—James Phillips, black seaman, aged twenty-five, born in Nova Scotia, his parents natives of St. Domingo, was admitted into the Marine Hospital, December 5th, 1855, having a slight enlargement of the left inguinal gland, for which the local application of tinct. iodine was directed, followed by pressure. This treatment was continued until the eleventh, when at my morning visit he exhibited *a half gallon of purely white urine*, voided during the past twenty-four hours. It was entirely free from urinous odor, having the smell and appearance of human breast milk. His history he gave as follows: In

December, 1854, he sailed from Liverpool to St. Domingo. During the voyage from the latter port to New York he was sick three weeks with fever, and pending convalescence he first perceived the milky appearance of his urine, which after a week's continuance disappeared. On his arrival at New York it returned, and abated on the third day. Left New York for Liverpool, (Nova Scotia,) and thence to Barbadoes, during which voyage he slept one night imprudently on deck, and was drenched by rain. This brought on a suppression of urine, which catheterism partially relieved. For several days subsequent he suffered much pain at each attempt to evacuate his bladder, discharging at such efforts coagulated shreds. He next sailed for St. Thomas, thence to Santa Cruz, where the milky urine again returned, and continued during his voyage to Denmark—never free from it longer than two or three days at a time. Two months previous to his arrival at Mobile, he contracted gonorrhœa, which was cured, and was succeeded by the return of the milky urine. Up to this date the largest amount of the urine discharged in twenty-four hours has been a half gallon. Throughout, it has chiefly prevailed at night, much the greater portion during the twenty-four hours being passed from bedtime to daylight. Singular to say, his appetite and digestion are always good, with far less emaciation than would have been looked for; skin dry; bowels ordinarily regular.

*Treatment.*—December 12th, 13th, 14th, 15th. Tinct. Ferri Chloridi, ten drops three times a day. Diet nutritious, abstinence from salt provisions.

17th.—From mid-day, yesterday, has passed *one hundred and twenty-four ounces,* (only four ounces less than one gallon,) which, upon rest, assumed the appearance of *blanc-mange.* ℞.—Infus. Cinchon Comp. ℥ij, thrice daily.

18th.—Quantity reduced to *half a gallon;* less turbid, and of less specific gravity; retains its fluidity, with few masses of albuminous shreds; slight odor of urea. Continue the infusion of cinchona.

19th.—Urine clear, quantity diminished. ℞.—Ferri Sulph., grs. iv, every eight hours; and at bedtime, Pulv. Doveri, grs xv.

20th.—Quantity increased, with milky appearance; inodorous, with flocculent shreds.

24th.—No material change until to-day; the urine coagulates. ℞.—Terebinth Venet., Ext. Gentian, āā ʒij; Ferri Sulph., Gum Kino, āā ʒss. M. Div. mass in pilul. āā grs. jss. S. Three thrice daily, and at bedtime Pilul. Rhei Compos., grs. v.

26th.—Continue treatment.

31st.—No change of urine; slight fever, puffiness of face, and swelling of cervical ganglia; complains of sore throat. These symptoms attributed to over-eating and exposure the preceding night unknown to the officers. Ol. Ricini, ℥j. Apply tincture of iodine to glands.

January 1st.—Fever last night, preceded by slight chill. ℞.—Sulph. Quiniæ, grs. x., to be repeated in six hours. Suspend pills.

3d.—Recommence the pills.

10th.—Fever last night. Sulph. Quiniæ, grs. x.

11th.—Continue pills.

12th.—Dry cups to lumbar region; no change.

13th.—Volatile liniment to lumbar region.

14th.—Bals. Copaiv., ʒvij; Spts. æth. Nit., ℥j; Sodæ Bicarb., ʒijss; Tinct. Cubebs, ℥ss; Tinct. Opii Camph., ʒvi; Sacc alb. and Gum Acac. q. s. Ft. mist. ℥vi. Habt. ℥ss, ter die.

15th, 16th, 17th.—Continue treatment. Under this course, the urine assumed a more clear and natural appearance, *three pints being passed in twenty-four hours.* With this stationary advantage he remained in the hospital until the twenty-third, when the vessel being about to sail for Europe, he was compelled to leave. On the 27th of November, 1858, I met the captain under whom Phillips sailed, and he reports him to be in Liverpool in perfect health and robust. His improvement commenced at the time of his discharge from the hospital and continued until he reached port.

*Remarks.*—Cases of this nature are rare, yet not unnoticed by systematic writers. They are recorded under the various titles of *chylous, milky and albuminous* urine, and appear to occur more particularly among natives, or residents of tropical regions. The pathology of the abnormal conversion of the urinary elements can hardly be considered decisive, notwithstanding the various resources by which chemistry has lent its aid to physiology. Nor have the various modes of treatment contributed to the solution. Works on general practice, from Morgagni to the present day, throw very little light upon the subject; hence every additional case recorded acquires peculiar interest. Among the writers who have referred to the disease in individual cases are Prout,* Elliotson,† Bonyun,‡ and Stedman.§ The last two have published each a specific case, with the mode of treatment. Dr. Golding Bird devotes a very short paragraph to the subject, and confines himself merely to two assertions—first, his incredulity that milk is ever detected in the urine, except when fraudulently introduced; secondly, that cases of milk-like urine

* Inquiry into the Nature of Calculus, Diabetes, etc.

† See Good's Study of Med., vol. ii. p. 625.

‡ Lond. Lancet, 1846, vol. iii. p. 253.

§ Amer. Jour. Med. Sciences, May, 1828, p. 295.

are those containing *phosphatic*, *purulent* or *fatty urine*. He admits, however, that some of the elements of milk may be present, as bile occasionally is, through vicarious action of the kidneys, and its presence established by the appearance of *casein*, as a principle dissolved in globules of fatty or oily matter. The subjection to the microscope of specimens discharged from my patient, (at different stages of his disease, and with the urine kept for shorter or longer periods after the evacuation,) leaves no doubt as to the fact that there is, under certain functional derangement, such an element in the human urine independent of any vicarious relation with utero-gestation.

In the *Amer. Jour. of the Med. Sciences* referred to above, Dr. George W. Stedman, of St. Croix, W. I., reports a case of "Milky Urine terminating fatally in Tubercular Consumption." The patient was a colored lad aged seventeen. The pathology of this patient varies very little from the case I have just related, but the specific action differs in the absence in my patient of those organic lesions which ultimately involved the lungs of Dr. Stedman's, through means of a general tubercular diathesis. While in Phillips there was no lesion of nutrition, in Dr. Stedman's patient a defective assimilation marked the case throughout, not only in the symptoms, but also in the fatal result following the depletory and mercurial course pursued in its treatment. His case, therefore, is more nearly allied to Dr. Good's paruria incocta, or unassimilated urine. Again, the largest quantity of urine passed by his patient in one day was fifty-five ounces, being sixty-nine ounces less than the largest amount passed by mine.

The case published by Dr. Bonyun occurred at Georgetown, British Guiana. The patient, a creole of Demarara, aged thirty-one, was attacked with the disease without any morbid prodrome, and while in the enjoyment of good health. The functional disturbances in this patient also differed from mine, especially in assimilative integrity; hence he is partly justified in classing it with Good's "paruria incocta," although I am not altogether satisfied that the analogy between *uncomplicated* cases of milky urine and that disease is strictly correct, there being very rarely, as far as reported cases of the former instruct us, such decided absence of assimilation of food or remedies, or the unchanged transit of drugs, etc., to the bladder, independent of chylopoietic action, as testified by the experiments of Home, Wallaston, Marcet, and others. Dr. Bonyun reports his case, however, for the particular purpose of referring to the curative influence of *rhizophora racemosa*, or mangrove, a tree indigenous to British Guiana, which remedy, after a failure with all others, was successfully prescribed by an old negress, in

*one-ounce doses of decoction of the bark, four times a day.* The patient, he says, was restored to health.

MICROSCOPY.—*Specimen* No. 1. Passed December 13th; examined 23d. Milk color; inodorous; covered apparently by a pellicle of cream, which, under the microscope, exhibited *milk and oil globules.* A portion taken from an inch below the surface contained coagulated grains of *casein.* The lower stratum (about one-sixteenth of the whole) was composed of mucus, and on evaporation afforded crystals of neutral triple phosphate of magnesia and ammonia.

*Specimen* No. 2. Passed December 14th, and, while fresh, tested with one-eighth sulphuric ether; examined by microscope on 24th. Covered with creamy pellicle, exhibiting *milk and oil globules*, some of the latter entire but most of them formed into minute drops. At the bottom the fluid contained milk and mucus globules, resembling the imperfect milk of menstruating females. No crystals formed by evaporation, except a few small ones of lithate of ammonia. A portion from the bottom of the bottle was concentrated by heat, and on the addition of acetic acid produced a separation of casein, about one-fourth of the whole. The subsident fluid contained mucus, but no crystals except the minute granules of amorphous powder of lithate of ammonia.

*Specimen* No. 3. Passed December 16th; examined 25th. The fluid was separated into three distinct strata. The upper one, three-fourths of an inch thick, cream colored, and, under the microscope, exhibited oil and milk globules, with casein coagulated into granules. The middle layer, one and a quarter inches thick, had the appearance of healthy urine, and when evaporated exhibited crystals of bibasic triple phosphate of magnesia and ammonia. No ammonia was used as a test. The lower stratum, half an inch thick, on evaporation yielded crystals of lithate and super-lithate of ammonia, with some needle-shaped crystals of urea.

*Specimen* No. 4. Passed December 19th; examined 31st. Opalescent; had the smell of putrid animal matter; slightly ammoniacal; surface covered with a slight pellicle in which very few oil globules were perceptible, but it was filled with a great quantity of animalculæ; the usual product of decomposed animal matter; a portion of the specimen taken from the centre of the bottle, by means of a pipette, exhibited, on evaporation, rectangular prisms of neutral phosphate of magnesia, and some crystals of chloride of sodium were apparent. On being concentrated, and nitric acid added, albuminous coagula were produced, and the subsident fluid afforded scales of nitrate of urea.

